# Rapid FAK Phosphorylation and Progressive Tight-Junction Protein Alterations Under Hyperosmotic Stress in Corneal Epithelial Cells

**DOI:** 10.3390/ijms27219716

**Published:** 2026-10-29

**Authors:** Yumeng Zhou, Chuanxue Ma, Ying Gao, Yujun Wang, Zeyu Zhang, Mingjia Shi, Yi Liu

**Affiliations:** Department of Ophthalmology, Nanjing Hospital of Chinese Medicine Affiliated to Nanjing University of Chinese Medicine, Nanjing 210022, China

**Keywords:** focal adhesion kinase, proline-rich tyrosine kinase 2, hyperosmotic stress, tight junction proteins, corneal epithelium, dry eye disease, PF-562271

## Abstract

Dry eye disease (DED) is characterized by tear hyperosmolality and ocular surface injury, but the contribution of focal adhesion kinase (FAK)-related signaling to tight junction (TJ) protein alterations remains unclear. We combined a scopolamine hydrobromide (SCOP)-induced desiccating stress mouse model with human corneal epithelial (HCE-T) cells exposed to NaCl-induced hyperosmolarity, together with transcriptomics, immunofluorescence, immunoblotting, pharmacological inhibition, and siRNA-mediated FAK knockdown. RNA sequencing identified 823 significant large-effect differentially expressed genes; analysis of the 3741 FDR-corrected significant genes revealed significant enrichment of focal adhesion, extracellular matrix–receptor, cell adhesion, and tight junction pathways. In HCE-T cells, hyperosmotic stress increased FAK Tyr397 phosphorylation as early as 1 h without changing total FAK. At 24 h, p-FAK declined while total FAK protein levels remained stable, consistent with dephosphorylation at Tyr397; by 48 h, total FAK protein was lost, accompanied by cell injury. TJ protein abundance and junctional continuity decreased progressively with increasing osmolarity and exposure duration. PF-562271, a dual FAK/Pyk2 inhibitor, produced a concentration-dependent reduction in FAK Tyr397 phosphorylation and, at 2.5 μM, also reduced Pyk2 Tyr402 phosphorylation while partially preserving ZO-1, claudin-1, and occludin abundance. FAK knockdown independently attenuated TJ protein loss and partially preserved ZO-1 junctional continuity under 500 mOsm/L hyperosmotic stress. These findings support a time-dependent contribution of FAK-related signaling to hyperosmotic stress-induced TJ protein remodeling.

## 1. Introduction

Dry eye disease (DED) is a widespread chronic ocular surface disorder characterized by tear-film instability, tear hyperosmolality, inflammatory responses, and corneal epithelial damage [1]. According to the TFOS DEWS II Epidemiology Report, the global prevalence of DED ranges from 5% to 50% and continues to rise [1]. Tear hyperosmolality is recognized as a core pathogenic driver that initiates cellular injury, including epithelial barrier dysfunction, inflammatory signaling, and apoptosis [2,3].

The corneal epithelial barrier relies on intact tight junctions composed of occludin, ZO-1, and claudin-1 [4,5]. Hyperosmotic stress disrupts tight-junction (TJ) structure through multiple pathways, including the TNF-α/MMP axis and IL-37/TNF-α/CTSS signaling [6,7]. However, the upstream molecular mechanisms coordinating cytoskeletal dynamics and tight-junction stability under hyperosmotic stress remain poorly defined.

Focal adhesion kinase (FAK) is a non-receptor tyrosine kinase that regulates cell adhesion, migration, and mechanotransduction [8,9,10]. Autophosphorylation at Tyr397 creates a binding site for Src-family kinases and is commonly used as a surrogate marker of FAK-related signaling, although measurement at this site alone does not constitute a complete kinase-activity assay [9,11]. Emerging evidence indicates that FAK interacts with tight-junction proteins and contributes to epithelial barrier integrity [12,13]. Hyperosmotic stress rapidly induces FAK Tyr397 phosphorylation in several cell types [14]. FAK also participates in osmoadaptive transcriptional programs, including regulation of the osmosensitive transcription factor TonEBP/NFAT5 under hypertonic conditions [15]. The role of FAK in epithelial barrier function appears context-dependent: in cadmium-induced testicular injury, a FAK/occludin/ZO-1 complex contributes to barrier disruption [12], whereas in intestinal epithelium, FAK-mediated tight-junction redistribution is protective [13]. This dichotomy underscores the need to define FAK function in a tissue- and stimulus-specific manner.

We hypothesized that hyperosmotic stress induces a time-dependent FAK Tyr397 phosphorylation response accompanied by progressive changes in TJ protein expression and structure. To this end, we combined exploratory transcriptome sequencing (RNA-seq) with imaging and immunoblotting experiments in HCE-T cells, interrogated FAK/Pyk2-sensitive signaling with PF-562271 at different concentrations (with concurrent measurement of Pyk2 Tyr402 phosphorylation), and used siRNA-mediated FAK knockdown to provide orthogonal genetic validation.

## 2. Results

### 2.1. Establishment and Validation of a SCOP-Induced Murine Dry Eye Model with Tear Hyperosmolality

**Figure 1:**
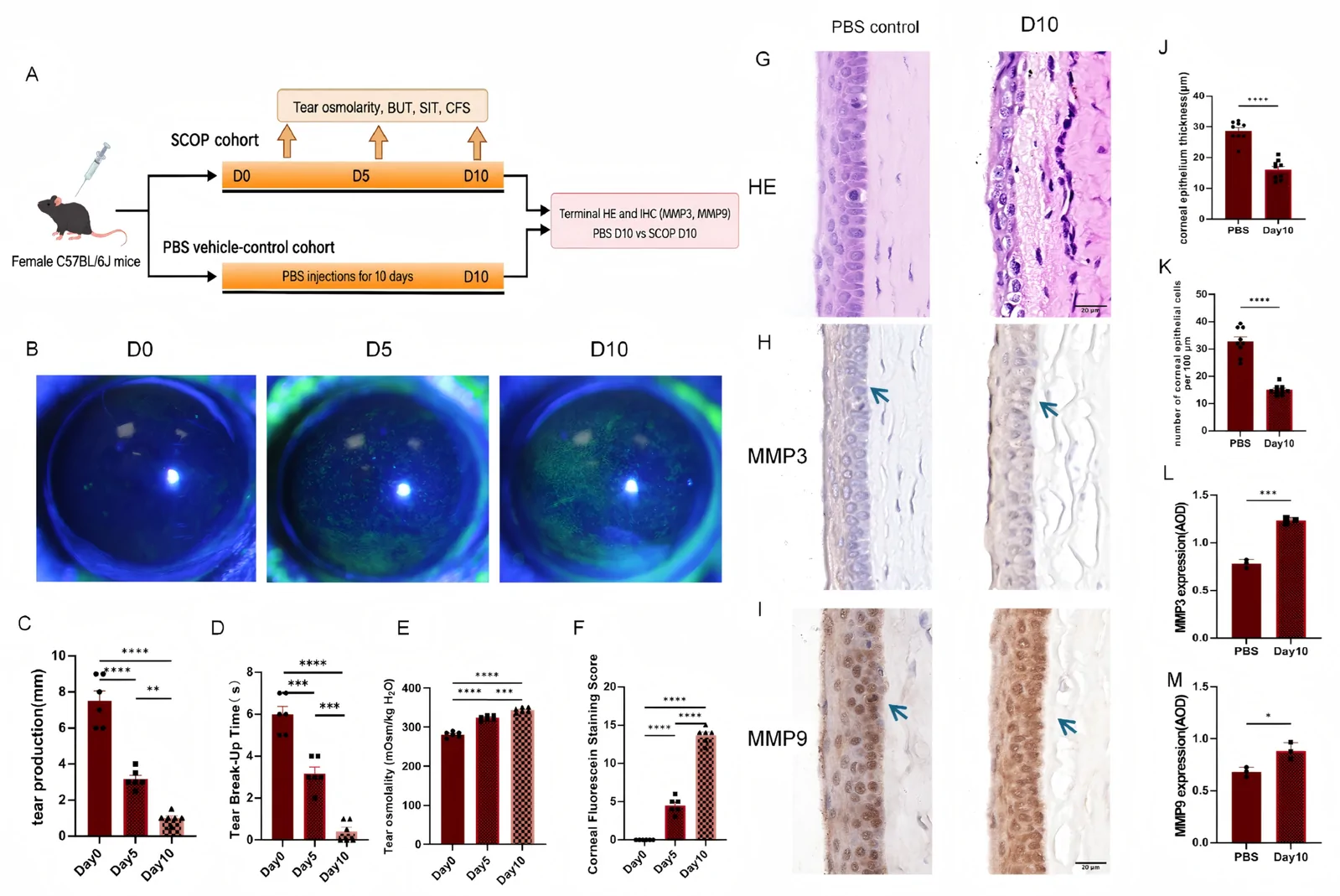
**Scopolamine hydrobromide (SCOP)-induced desiccating-stress mouse model.** (**A**) Experimental workflow. The SCOP cohort was assessed longitudinally at the pre-SCOP baseline (Day 0) and after 5 and 10 days of SCOP exposure. A separate vehicle-control cohort received volume- and schedule-matched phosphate-buffered saline (PBS) injections for 10 days and was used for terminal tissue comparisons. (**B**) Representative corneal fluorescein staining (CFS) images from the SCOP cohort at Day 0 (baseline), Day 5, and Day 10. (**C**–**F**) Tear production, tear-film break-up time, tear osmolality, and CFS score. Each point represents one mouse (tear production, tear-film break-up time, and CFS score averaged over both eyes; tear samples from both eyes pooled for osmolality); the same six mice in the SCOP cohort were measured longitudinally at Days 0, 5, and 10 (*n* = 6 mice); these mice were included among the nine SCOP mice used for terminal hematoxylin and eosin (H&E) analysis. Longitudinal outcomes were analyzed using repeated-measures one-way ANOVA followed by Tukey’s multiple-comparisons test. Panels G–M show terminal comparisons between the Day 10 PBS vehicle-control cohort and the Day 10 SCOP cohort. (**G**) Representative hematoxylin–eosin staining. (**H**,**I**) Representative matrix metalloproteinase 3 (MMP3) and matrix metalloproteinase 9 (MMP9) staining; blue arrows indicate representative positively stained regions; scale bars, 20 μm. (**J**,**K**) Corneal epithelial thickness and epithelial-cell number per 100 μm; nine right-eye corneas from nine mice were analyzed per condition (*n* = 9 corneas/group). (**L**,**M**) MMP3 and MMP9 average optical density (AOD); three right-eye corneas from three mice were analyzed per condition (*n* = 3 corneas/group); these mice were a subset of the nine mice used for H&E in each condition. Cornea-level summaries were compared by two-sided unpaired *t* test. Individual symbols represent individual mice or biological replicates, as specified; bars show the mean ± standard error of the mean (SEM). * *p* < 0.05, ** *p* < 0.01, *** *p* < 0.001, and **** *p* < 0.0001.

To establish the scopolamine (SCOP)/desiccating-stress model, longitudinal ocular-surface measurements were obtained from both eyes of each mouse at baseline and after 5 and 10 days of SCOP exposure (Figure 1A). The same six mice contributed to each longitudinal time point. Corneal fluorescein staining (CFS) was minimal at the pre-SCOP baseline (Day 0), increased at Day 5, and was most extensive at Day 10 (Figure 1B).

The longitudinal data showed progressive ocular-surface changes: tear production and tear-film break-up time decreased, whereas tear osmolality and CFS scores increased from Day 0 to Day 10 (Figure 1C–F). Estimated tear osmolality rose from 280.8 ± 3.0 mOsm/kg H_2_O at Day 0 to 323.3 ± 2.5 mOsm/kg H_2_O at Day 5 and 343.3 ± 2.5 mOsm/kg H_2_O at Day 10 (mean ± SEM, *n* = 6 mice; Figure 1E). Both eyes were measured at each session: tear production, tear-film break-up time, and CFS scores were averaged within each mouse, and tears from both eyes were pooled into one sample for osmolality measurement; the mouse was the experimental unit for these repeated measurements.

At Day 10, SCOP mice showed reduced corneal epithelial thickness and cell number (H&E, *n* = 9 mice/condition) and increased MMP3/MMP9 immunoreactivity (IHC, *n* = 3 mice/condition; a subset of the same nine mice; Figure 1G–M). Together with the longitudinal findings, these changes establish an ocular-surface phenotype and provide context for the exploratory whole-eye transcriptomics.

### 2.2. Whole-Eye Transcriptomics Identifies Adhesion- and Tight-Junction-Related Programs

**Figure 2:**
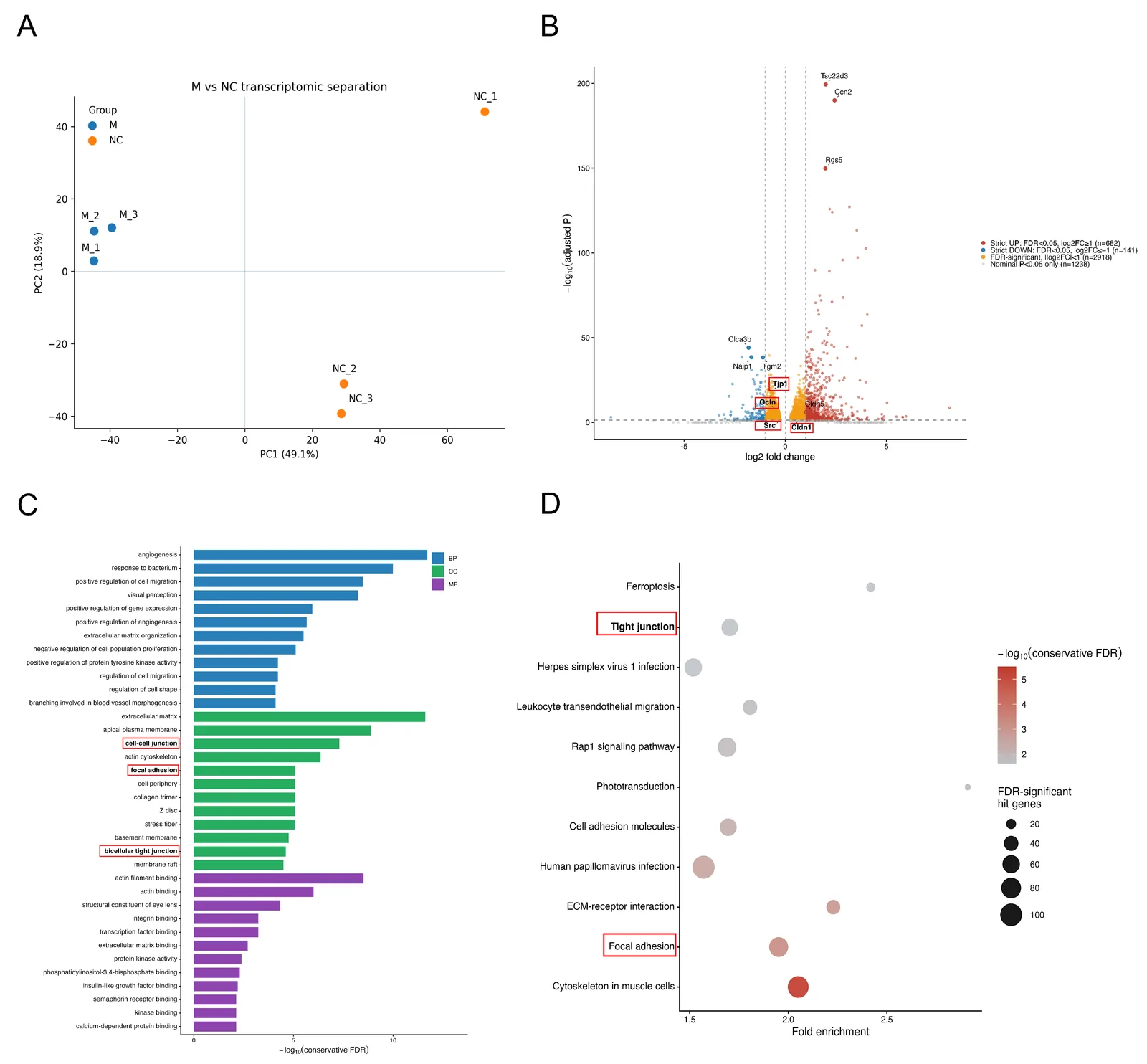
Whole-eye transcriptomic overview in SCOP/desiccating-stress model (M) and PBS vehicle-control (NC) mice; both whole eyes from each mouse were pooled within mouse to generate one biological replicate (*n* = 3 mice/group). (**A**) Principal-component analysis of sample-level separation. (**B**) Volcano plot of M versus NC differential expression. Strict large-effect genes were defined as FDR < 0.05 and |log_2_FC| ≥ 1 (682 upregulated and 141 downregulated); 2918 additional genes were FDR-significant with |log_2_FC| < 1, and 1238 genes were nominally significant only (nominal *p* < 0.05 and FDR ≥ 0.05). *Tjp1*, *Ocln*, *Src*, *Cldn1*, and *Cldn5* are annotated. (**C**) GO enrichment among all FDR-significant genes (*n* = 3741), grouped by biological process, cellular component, and molecular function. (**D**) KEGG enrichment in the same FDR-significant foreground. In (**C**), bar length represents −log_10_(conservative FDR), whereas color denotes GO domain (BP, CC, or MF). In (**D**), color represents −log_10_(conservative FDR), bubble size represents the number of hit genes, and the x-axis shows fold enrichment. Tight-junction- and focal-adhesion-related terms are outlined for orientation.

We next profiled whole-eye transcriptomes in the SCOP/desiccating-stress model. Both whole eyes from each model (M group) or PBS vehicle-control (NC group) mouse were pooled within each mouse to generate one biological replicate (*n* = 3 mice/group) and subjected to RNA sequencing. Principal-component analysis separated the M and NC transcriptomes (Figure 2A). For gene-level reporting, strict differentially expressed genes (DEGs) were defined as FDR < 0.05 and |log_2_FC| ≥ 1, yielding 823 genes (682 upregulated and 141 downregulated; Figure 2B).

Several canonical TJ-associated genes—*Tjp1* (log_2_FC = −0.68, FDR = 1.9 × 10^−18^), *Ocln* (−0.45, FDR = 1.2 × 10^−7^), *Src* (−0.34, FDR = 3.1 × 10^−4^), and *Cldn1* (+0.37, FDR = 5.0 × 10^−3^)—remained FDR-significant but fell below the large-effect cutoff and were therefore treated as FDR-significant moderate-effect observations rather than strict DEGs (Figure 2B and Figure S1C; Table S1); *Ptk2* did not meet the differential-expression criteria.

For pathway-level analysis, all genes meeting FDR < 0.05 were analyzed irrespective of fold-change magnitude (*n* = 3741), retaining coordinated moderate-effect changes under gene-level FDR control. GO analysis identified extracellular-matrix organization and protein-tyrosine-kinase regulation among biological processes; extracellular matrix, cell–cell junction, focal adhesion, basement membrane, and bicellular tight junction among cellular components; and integrin/ECM binding and kinase-related functions among molecular functions (Figure 2C). KEGG analysis independently identified focal adhesion, ECM–receptor interaction, cell-adhesion molecules, and tight junction among the robust pathways (Figure 2D); the contributing FDR-significant focal-adhesion and tight-junction genes are listed in Table S1.

Whether this pathway signal depended on the effect-size criterion was examined directly. KEGG Tight junction was not over-represented among the 823 strict large-effect genes but was robustly enriched in the FDR-controlled 3741-gene foreground (conservative FDR = 0.0229; 53 hits; Figure S1A), 48 of which had |log_2_FC| < 1, including *Tjp1*, *Ocln*, *Src*, and *Cldn1*; GO bicellular tight junction showed the same threshold-dependent pattern (conservative FDR = 2.46 × 10^−5^; 40 hits). Thus, the TJ signal was distributed across coordinated moderate-effect changes rather than concentrated in a few large-effect DEGs. Hypothesis-driven gene-set analyses provided complementary support: an independently defined epithelial TJ core showed mixed remodeling (mROAST FDR = 0.0090), a significant downward component (mROAST FDR = 0.0201), and CAMERA support (*p* = 0.001138; Figure S1B), consistent with heterogeneous TJ remodeling with a predominantly downward epithelial-core component.

The unbiased analysis also returned visual-perception, phototransduction, photoreceptor, and lens-associated terms, as expected from the mixed tissue composition of pooled whole-eye samples; these terms were retained in the complete results but were not interpreted as corneal epithelial biology. In a sensitivity analysis excluding a prespecified high-specificity retinal/RPE/lens marker set from the pathway foreground, phototransduction and lens-structural enrichment became non-significant and visual-perception signals were markedly attenuated, whereas the focal-adhesion, ECM, cell-adhesion, and tight-junction programs remained robust (Figure S1D).

### 2.3. Hyperosmotic Stress Alters HCE-T Cell Morphology, Viability, and Wound Closure

**Figure 3:**
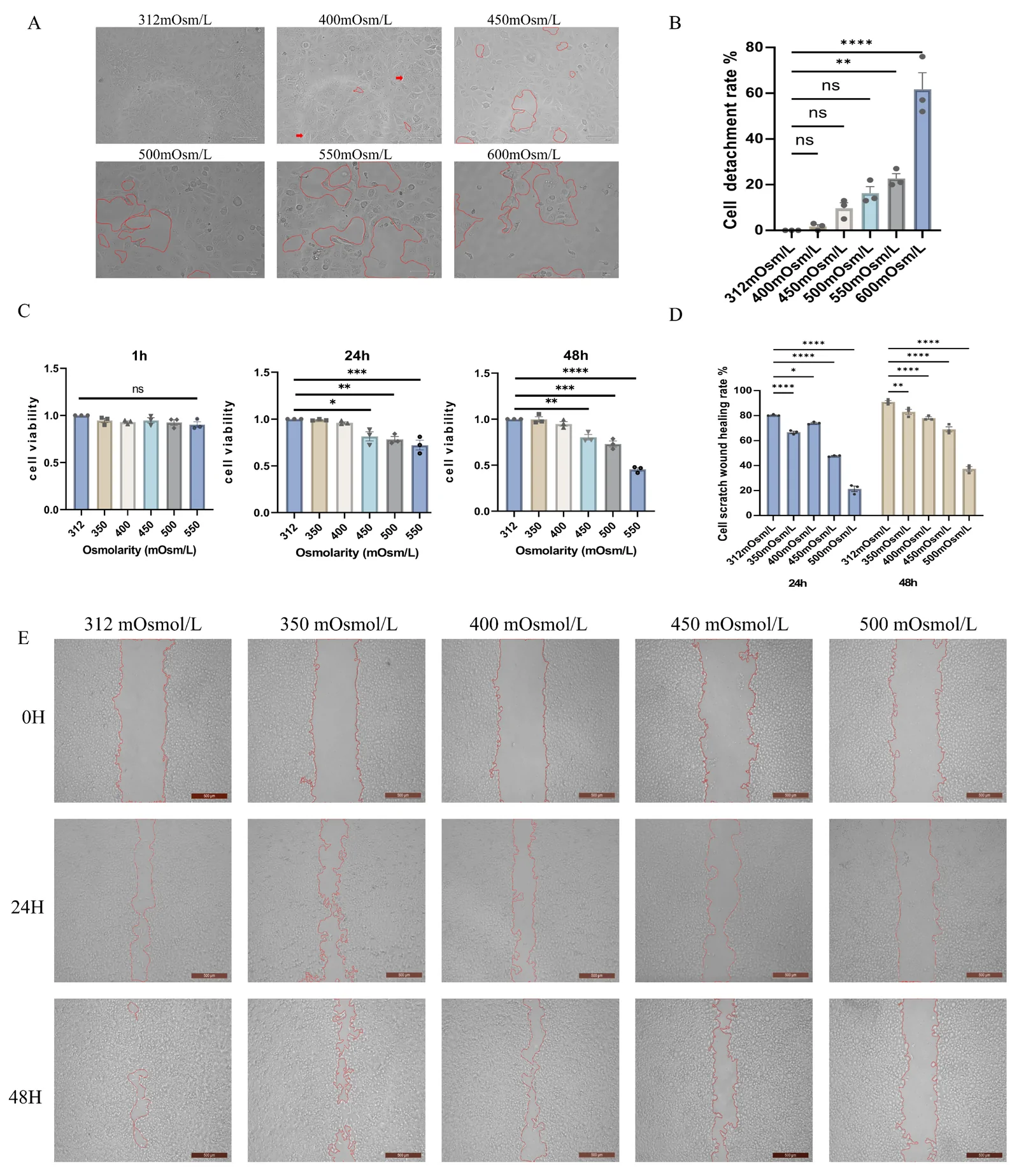
**Hyperosmotic stress alters human corneal epithelial (HCE-T) cell morphology, metabolic viability, and wound closure.** (**A**) Representative morphology at 312, 400, 450, 500, 550, and 600 mOsm/L. Red outlines indicate detached regions, and red arrows indicate representative cell-detachment sites. (**B**) Cell-detachment rate. (**C**) Cell Counting Kit-8 (CCK-8) signal, normalized to the 312 mOsm/L condition at the corresponding time, after 1, 24, and 48 h (312–550 mOsm/L). (**D**) Scratch-wound closure at 24 and 48 h (312–500 mOsm/L). (**E**) Representative scratch-wound images at 0, 24, and 48 h. Three independent experiments were performed; technical replicates or fields were averaged to generate one value per experiment (*n* = 3). Within each time point, groups were analyzed using one-way ANOVA followed by Tukey’s multiple-comparisons test. Colors and fill patterns distinguish osmolarity groups; individual symbols represent independent biological experiments; bars show the mean ± standard error of the mean (SEM). * *p* < 0.05, ** *p* < 0.01, *** *p* < 0.001, and **** *p* < 0.0001; ns, not significant. Scale bars, 125 μm (**A**) and 500 μm (**E**).

To examine the effects of increasing culture-medium osmolarity on HCE-T cells, we assessed morphology and detachment over 312–600 mOsm/L, CCK-8 metabolic activity over 312–550 mOsm/L, and wound closure over 312–500 mOsm/L (Figure 3).

Cells retained a closely apposed polygonal appearance at 312 mOsm/L, whereas cell shrinkage, intercellular gaps, and detachment increased as osmolarity rose (Figure 3A). Quantified detachment remained relatively low through 500 mOsm/L and was greatest at 550 and 600 mOsm/L (Figure 3B). Osmolarities of 450–600 mOsm/L are high experimental stresses relative to typical clinical tear values; 550–600 mOsm/L were used only to define the upper tolerance range, whereas mechanistic imaging and immunoblot experiments were restricted to ≤500 mOsm/L.

The normalized CCK-8 signal was not detectably reduced after 1 h. At 24 h, reductions emerged at 450–550 mOsm/L, and at 48 h the decline was greater, particularly at 500 and 550 mOsm/L (Figure 3C).

Hyperosmotic exposure also reduced scratch-wound closure at 24 and 48 h, with the largest reduction at 500 mOsm/L (Figure 3D,E). As proliferation was not inhibited during the assay, wound closure reflects the combined contribution of cell migration and proliferation and was not interpreted as migration alone.

### 2.4. Hyperosmotic Stress Alters Corneal Epithelial Tight-Junction Protein Organization in a Time-Dependent Manner

To assess the effects of hyperosmotic stress on tight-junction organization, immunofluorescence staining was used to examine the distribution of ZO-1 and claudin-1 in HCE-T cells.

At 312 mOsm/L, both proteins showed predominantly continuous junctional staining along cell borders. Similar patterns were observed after 1 h across the tested osmolarities, although localized weakening and discontinuity became apparent at higher osmolarities.

**Figure 4:**
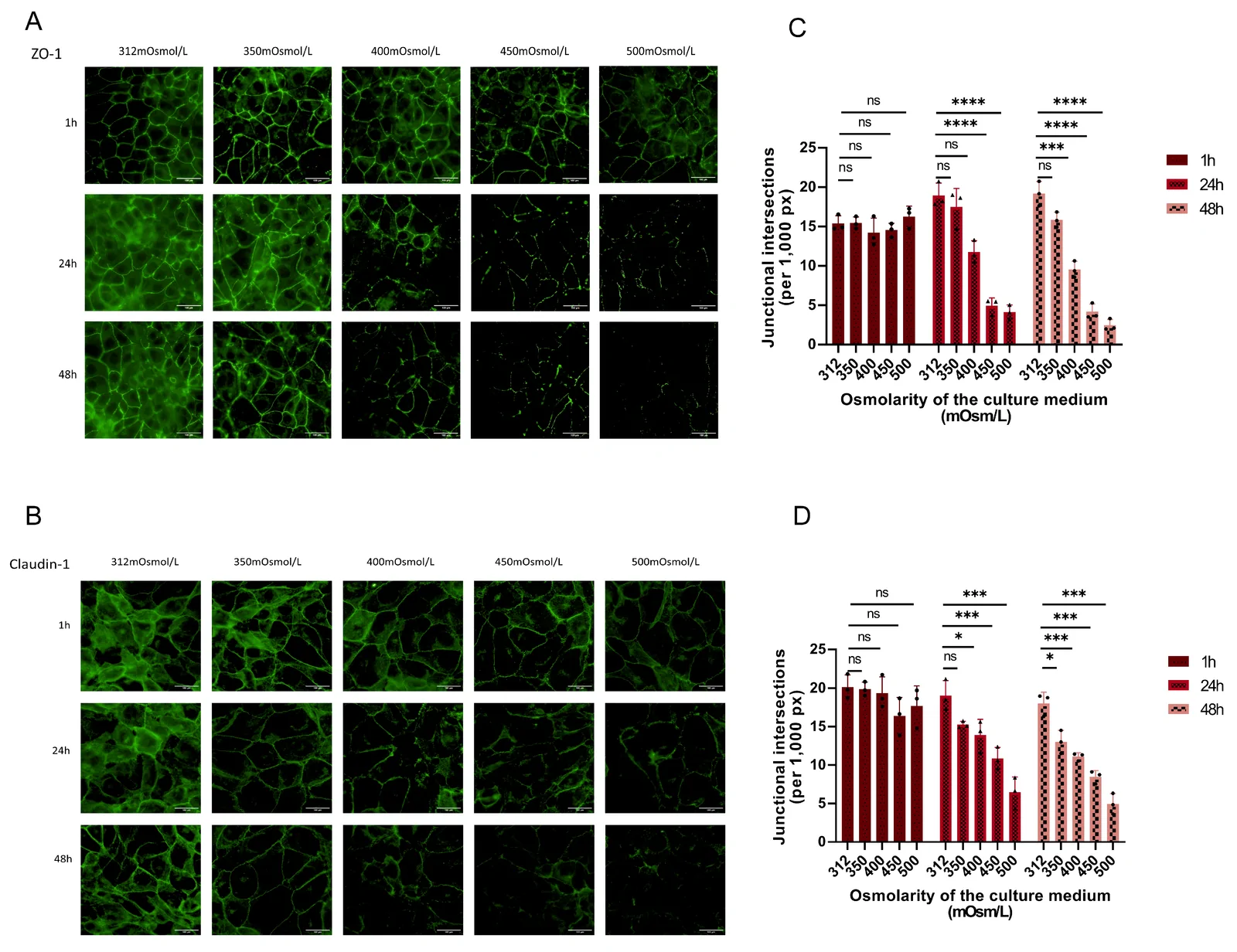
**Hyperosmotic stress alters zonula occludens-1 (ZO-1) and claudin-1 junctional organization in human corneal epithelial (HCE-T) cells.** (**A**,**B**) Representative single-channel images of ZO-1 (**A**) and claudin-1 (**B**) after exposure to 312, 350, 400, 450, or 500 mOsm/L medium for 1, 24, or 48 h; target-associated fluorescence is shown in green. (**C**,**D**) Junctional intersections per 1000 contour pixels for ZO-1 (**C**) and claudin-1 (**D**). Three non-overlapping fields were analyzed per condition in each independent experiment and averaged to one experiment-level value (*n* = 3 independent experiments). Within each time point, groups were analyzed using one-way ANOVA followed by Tukey’s multiple-comparisons test. Colors and fill patterns distinguish time points; individual symbols represent independent biological experiments; bars show the mean ± standard error of the mean (SEM). * *p* < 0.05, *** *p* < 0.001, and **** *p* < 0.0001; ns, not significant. Scale bars, 100 μm.

After 24 and 48 h, increasing osmolarity was associated with progressive fragmentation and loss of continuous junctional staining. These changes were most pronounced at 450 and 500 mOsm/L. Quantification of junctional intersections per 1000 contour pixels showed a corresponding reduction in junctional organization for both ZO-1 and claudin-1 (Figure 4C,D).

These findings show that sustained hyperosmotic stress reduces the continuity of ZO-1- and claudin-1-associated junctional staining. This imaging endpoint reflects altered TJ protein organization and does not directly measure transepithelial barrier function.

### 2.5. Per-Nucleus Immunofluorescence Shows Time-Dependent Changes in FAK and p-FAK

**Figure 5:**
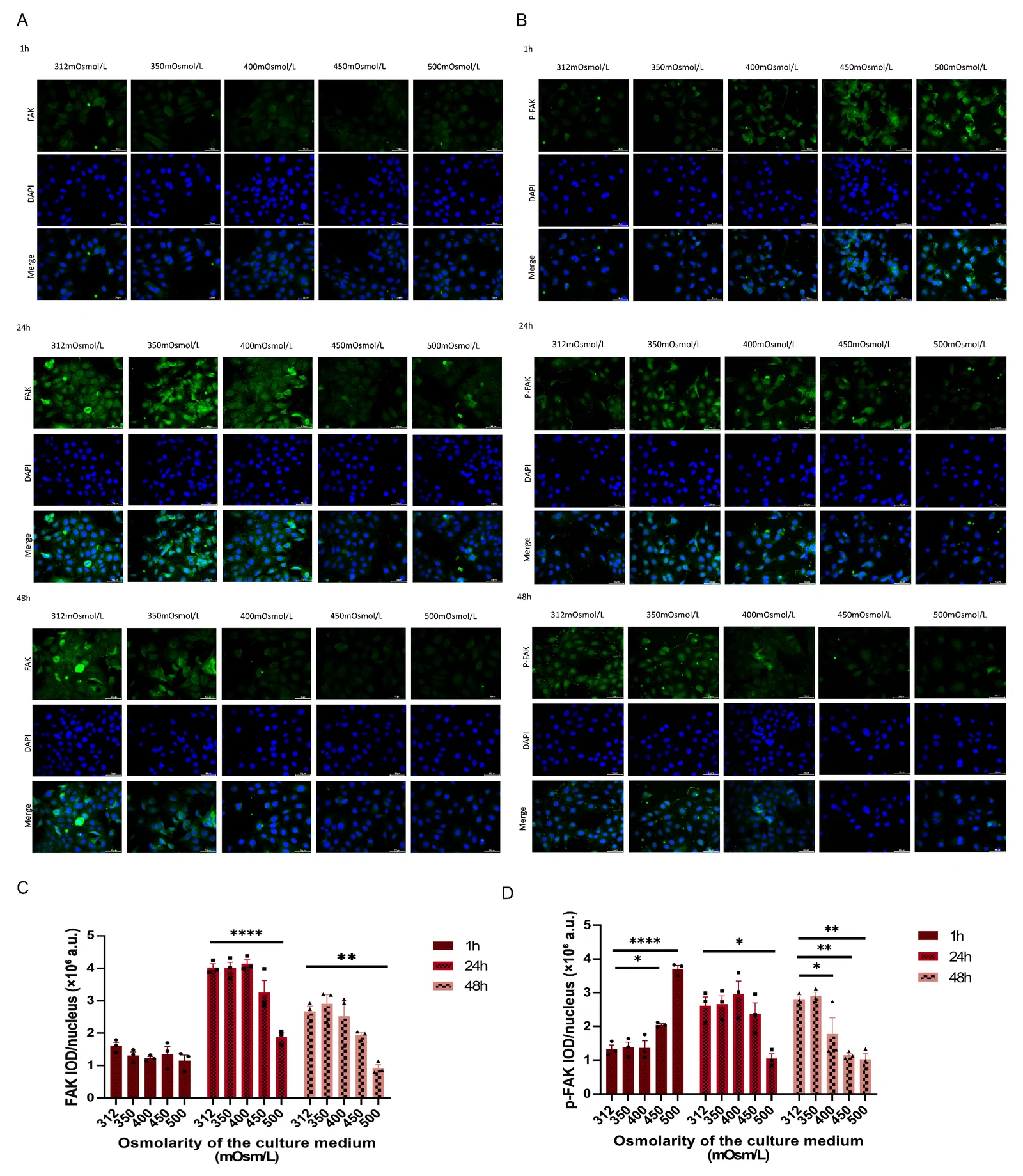
**Per-nucleus abundance and distribution of focal adhesion kinase (FAK) and phosphorylated FAK (p-FAK) in human corneal epithelial (HCE-T) cells under hyperosmotic stress.** (**A**,**B**) Representative wide-field immunofluorescence images of total FAK (**A**) and p-FAK Tyr397 (**B**) after treatment with 312–500 mOsm/L medium for 1, 24, or 48 h (green, target protein; blue, 4′,6-diamidino-2-phenylindole [DAPI]). (**C**,**D**) Background-corrected total FAK integrated optical density (IOD) per DAPI-positive nucleus (**C**) and p-FAK IOD per DAPI-positive nucleus (**D**). Three non-overlapping fields were averaged to one value per independent experiment (*n* = 3). Images were acquired with identical settings within each marker/time-point batch; because time points were stained and imaged separately, comparisons were restricted to osmolarity groups within each time point. Groups were analyzed using one-way ANOVA followed by Tukey’s multiple-comparisons test. Colors and fill patterns distinguish time points; individual symbols represent independent biological experiments; bars show the mean ± standard error of the mean (SEM). * *p* < 0.05, ** *p* < 0.01, and **** *p* < 0.0001. Scale bars, 50 μm.

Total FAK and Tyr397-phosphorylated FAK (p-FAK) were examined by immunofluorescence after 1, 24, or 48 h of hyperosmotic exposure (Figure 5A,B). For each field, background-corrected integrated optical density (IOD) was divided by the number of DAPI-positive nuclei; experiment-level values were generated by averaging fields (Figure 5C,D). No immunofluorescence p-FAK/FAK ratio was calculated because total FAK and p-FAK were measured on separate coverslips.

At 1 h, total FAK IOD per nucleus varied little across the tested osmolarities, whereas p-FAK IOD per nucleus increased at higher osmolarities, with the largest signals at 450–500 mOsm/L (Figure 5C,D). These data are consistent with an early increase in Tyr397 phosphorylation.

Within the 24 and 48 h imaging batches, high osmolarity was associated with lower total FAK and p-FAK IOD per nucleus, particularly at 500 mOsm/L (Figure 5C,D). The representative images show reduced signal extent under the highest osmolarities.

The control IOD values differed among time points because samples were processed and imaged in separate time-specific staining sessions. Exposure and gain were held constant within each marker and time point, and statistical comparisons were therefore restricted to osmolarity groups within the same time point; absolute IOD values were not compared across 1, 24, and 48 h. Per-nucleus normalization reduces confounding by cell number but does not eliminate other modality-specific sources of variation.

### 2.6. Immunoblotting Shows an Early FAK Phosphorylation Response and Progressive TJ Protein Loss

**Figure 6:**
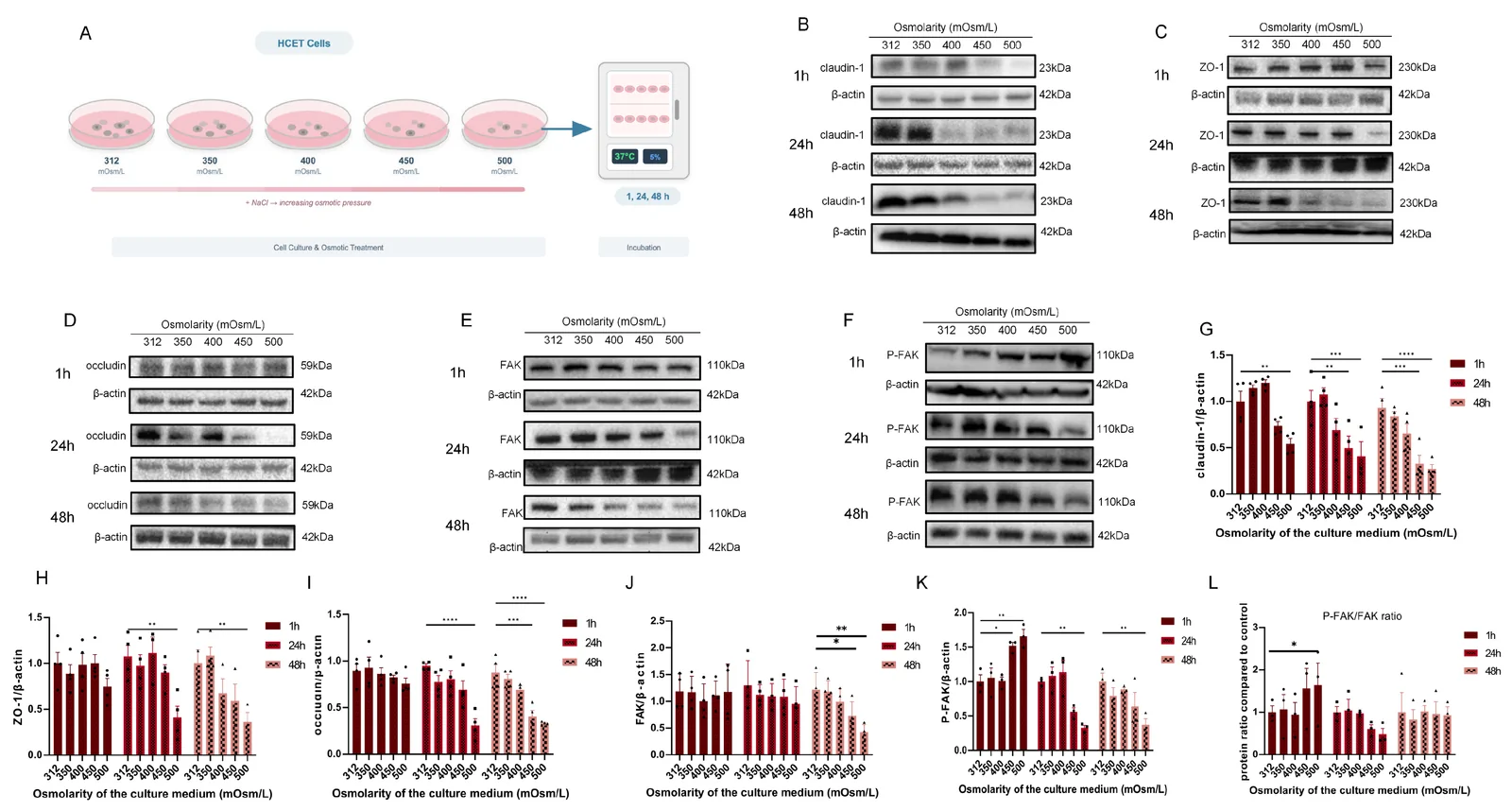
**Immunoblot assessment of tight-junction (TJ) proteins and focal adhesion kinase (FAK) signaling** during hyperosmotic exposure. (**A**) Experimental workflow. (**B**–**F**) Representative blots for claudin-1 (**B**), ZO-1 (**C**), occludin (**D**), total FAK (**E**), and phosphorylated FAK (p-FAK) Tyr397 (**F**) at 1, 24, and 48 h. (**G**–**K**) Densitometric values normalized to β-actin for claudin-1, ZO-1, occludin, total FAK, and p-FAK. (**L**) p-FAK/total FAK ratio relative to the 312 mOsm/L control within each time point. Individual symbols represent independent biological experiments; bars show the mean ± standard error of the mean (SEM; *n* = 3). Within each time point, groups were analyzed using one-way ANOVA followed by Tukey’s multiple-comparisons test. * *p* < 0.05, ** *p* < 0.01, *** *p* < 0.001, and **** *p* < 0.0001; ns, not significant.

Immunoblotting was used to evaluate claudin-1, ZO-1, occludin, total FAK, and p-FAK across 312–500 mOsm/L at 1, 24, and 48 h (Figure 6A–F); densitometric results are shown in Figure 6G–L.

At 1 h, total FAK remained comparatively stable, whereas p-FAK and the p-FAK/total FAK ratio increased at high osmolarity, supporting an early Tyr397 phosphorylation response (Figure 6J–L). Claudin-1 was already reduced at 500 mOsm/L, whereas ZO-1 and occludin showed smaller early changes (Figure 6G–I).

At 24 h, p-FAK abundance declined at high osmolarity while total FAK remained comparatively preserved. The p-FAK/total FAK ratio showed a downward pattern that did not reach significance in this dose–time-course experiment. By 48 h, total FAK itself was reduced under the highest osmolarities, and p-FAK declined in parallel; the ratio was consequently less informative at this later stage. Claudin-1, ZO-1, and occludin showed progressively lower abundance with increasing osmolarity and exposure duration (Figure 6G–I).

Taken together, hyperosmotic exposure produced a two-phase late FAK response: at 24 h, p-FAK decreased while total FAK was preserved, a pattern compatible with reduced Tyr397 phosphorylation, and by 48 h total FAK itself declined together with the broader protein loss and cell injury.

### 2.7. PF-562271 Suppresses FAK/Pyk2 Phosphorylation and Partially Preserves TJ Proteins

**Figure 7:**
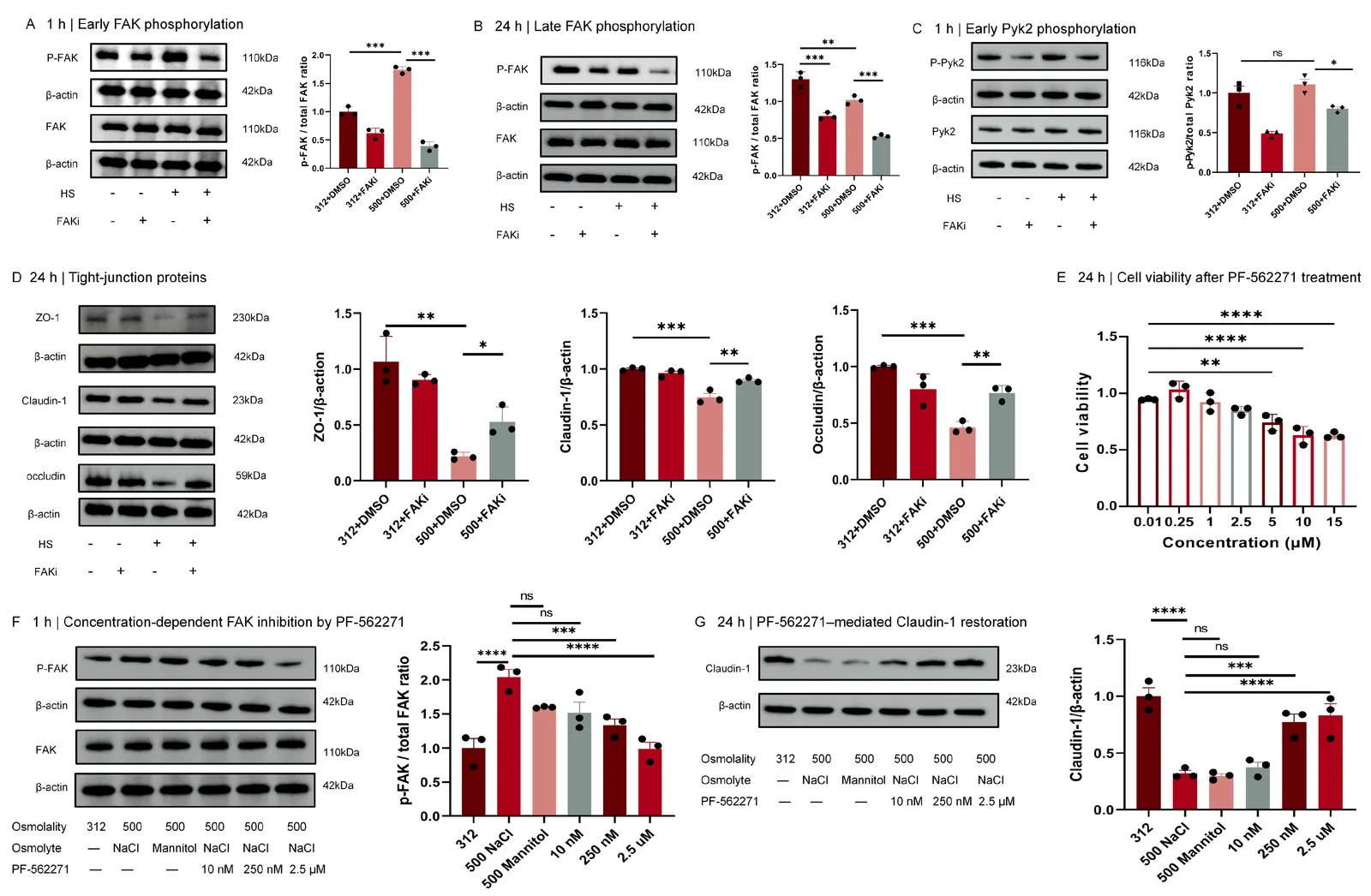
Pharmacological evaluation of PF-562271. For the four-group experiments, human corneal epithelial (HCE-T) cells were pretreated for 1 h with 2.5 μM PF-562271 or vehicle and then maintained in 312 or 500 mOsm/L medium with the corresponding treatment. (**A**) Total focal adhesion kinase (FAK), phosphorylated FAK (p-FAK) Tyr397, and the p-FAK/FAK ratio at 1 h. (**B**) Corresponding measurements at 24 h. (**C**) Total proline-rich tyrosine kinase 2 (Pyk2), phosphorylated Pyk2 (p-Pyk2) Tyr402, and the p-Pyk2/Pyk2 ratio at 1 h. (**D**) ZO-1, claudin-1, and occludin abundance at 24 h. (**E**) Cell Counting Kit-8 (CCK-8) metabolic viability signal after 24 h exposure to 0.01, 0.25, 1, 2.5, 5, 10, or 15 μM PF-562271. (**F**) p-FAK/FAK after 1 h in 312 mOsm/L medium, 500 mOsm/L NaCl medium, osmolality-matched 500 mOsm/L mannitol medium, or 500 mOsm/L NaCl medium containing 10 nM, 250 nM, or 2.5 μM PF-562271. (**G**) Claudin-1 abundance in the same groups after 24 h. FAKi in the panel labels denotes PF-562271 and does not imply FAK selectivity; HS, hyperosmotic stress. Colors and fill patterns distinguish treatment groups; individual symbols represent independent biological experiments; bars show the mean ± standard error of the mean (SEM; *n* = 3) and were analyzed using one-way ANOVA followed by Tukey’s multiple-comparisons test. * *p* < 0.05, ** *p* < 0.01, *** *p* < 0.001, and **** *p* < 0.0001; ns, not significant.

PF-562271 was evaluated as a dual FAK/Pyk2 inhibitor rather than as an FAK-selective probe (Figure 7). At 24 h, PF-562271 partially preserved ZO-1, claudin-1, and occludin abundance under 500 mOsm/L stress (Figure 7D). The ZO-1 reduction under 500 mOsm/L stress with vehicle was consistent with the significant 24 h ZO-1 decline in the independent dose–time-course experiment (Figure 6H). The 24 h PF-562271 concentration screen showed a progressive reduction in CCK-8 signal at higher concentrations (Figure 7E); balancing target-inhibition efficacy and cellular tolerance, 2.5 μM was selected as the working concentration for the subsequent mechanistic experiments.

At 1 h, 500 mOsm/L increased the p-FAK/total FAK ratio, and 2.5 μM PF-562271 reduced this ratio under both isotonic and hyperosmotic conditions (Figure 7A). At 24 h, the ratio was lower at 500 than at 312 mOsm/L and was further reduced by PF-562271 (Figure 7B), whereas total FAK remained comparatively preserved. PF-562271 also reduced Pyk2 Tyr402 phosphorylation; notably, 500 mOsm/L alone did not significantly increase the p-Pyk2/total Pyk2 ratio (Figure 7C).

In the concentration-response experiment, 500 mOsm/L NaCl and osmolality-matched mannitol produced similar early increases in the p-FAK/FAK ratio and similar 24 h reductions in claudin-1, supporting an osmotic component. Ten nanomolar PF-562271 had little effect, whereas 250 nM and 2.5 μM produced greater p-FAK suppression and claudin-1 preservation (Figure 7F,G). These data support PF-562271-sensitive FAK/Pyk2 signaling.

### 2.8. FAK Knockdown Partially Attenuates Hyperosmotic-Stress-Associated TJ Protein Loss

**Figure 8:**
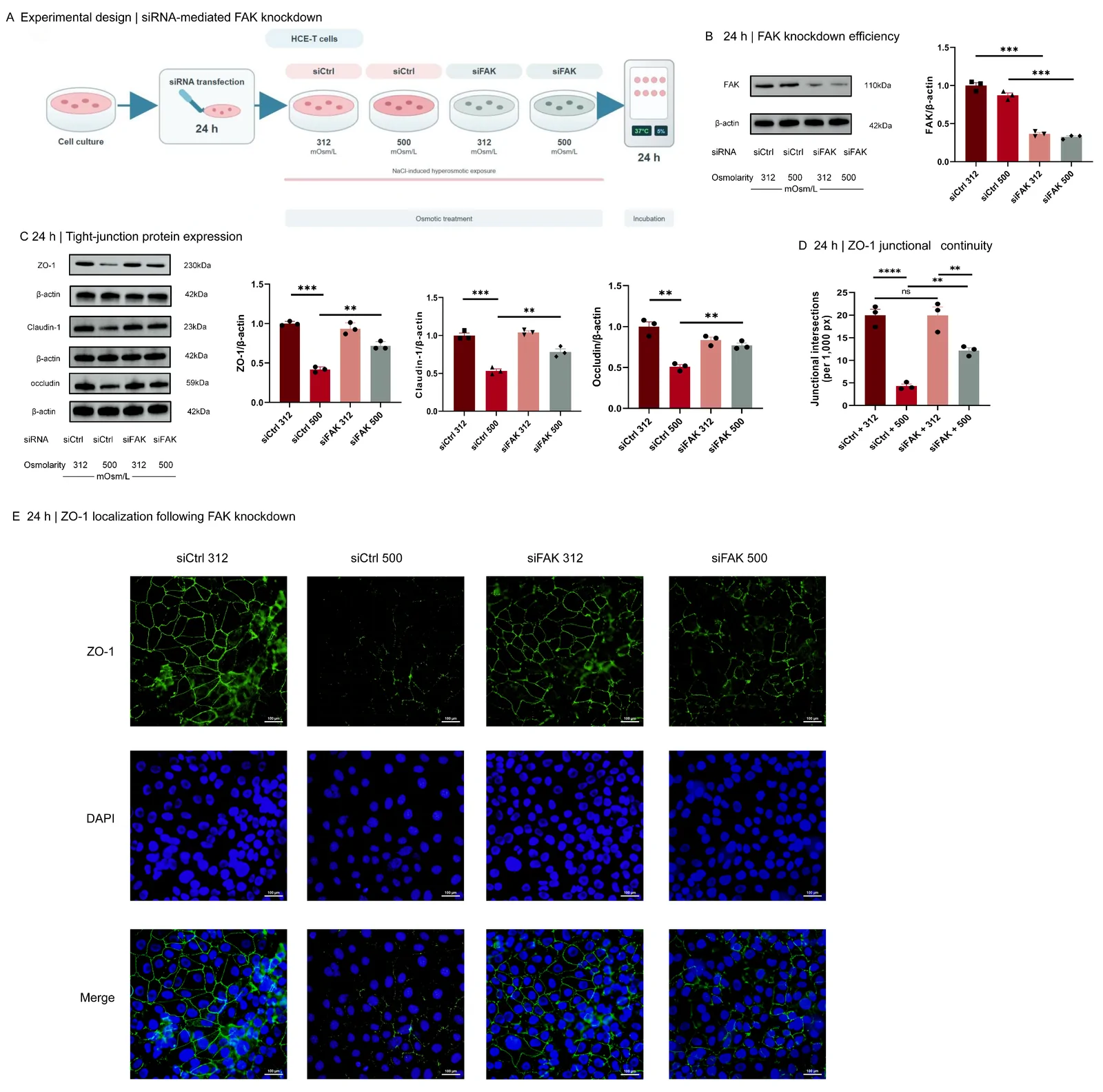
**Small interfering RNA (siRNA)-mediated focal adhesion kinase (FAK) knockdown partially attenuates tight-junction (TJ) protein loss** under hyperosmotic stress. The FAK-targeting duplex was hFAK si-1 (siFAK1; labeled siFAK in the panels). (**A**) Experimental design: human corneal epithelial (HCE-T) cells were transfected with non-targeting control siRNA (siCtrl) or siFAK1 for 24 h and then exposed to 312 or 500 mOsm/L medium for 24 h. (**B**) Representative total FAK immunoblot and densitometry confirming knockdown. (**C**) Representative ZO-1, claudin-1, and occludin immunoblots and densitometry. (**D**) Quantification of ZO-1 junctional intersections per 1000 contour pixels. (**E**) Representative zonula occludens-1 (ZO-1; green), 4′,6-diamidino-2-phenylindole (DAPI; blue), and merged images; scale bars, 100 μm. Three non-overlapping fields were averaged to one value per independent experiment for imaging analysis. Colors and fill patterns distinguish treatment groups; individual symbols represent independent biological experiments; bars show the mean ± standard error of the mean (SEM; *n* = 3) and were analyzed using one-way ANOVA followed by Tukey’s multiple-comparisons test. ** *p* < 0.01, *** *p* < 0.001, and **** *p* < 0.0001; ns, not significant.

To provide an orthogonal genetic test, HCE-T cells were transfected with control siRNA (siCtrl) or hFAK si-1 (siFAK1; shown as siFAK in Figure 8) for 24 h and then exposed to 312 or 500 mOsm/L medium for an additional 24 h (Figure 8A). siFAK1 reduced total FAK under both osmolarities, confirming knockdown (Figure 8B). Under 500 mOsm/L stress, siFAK1 increased ZO-1, claudin-1, and occludin abundance relative to siCtrl, although the values did not uniformly return to the corresponding isotonic condition (Figure 8C).

ZO-1 imaging showed severe loss of continuous junctional staining in siCtrl cells at 500 mOsm/L. FAK knockdown partially preserved ZO-1-positive junctional intersections under the same stress, while siFAK did not significantly alter junctional continuity at 312 mOsm/L (Figure 8D,E). These results support a contribution of FAK to the TJ protein response but do not demonstrate functional barrier recovery.

## 3. Discussion

**Figure 9:**
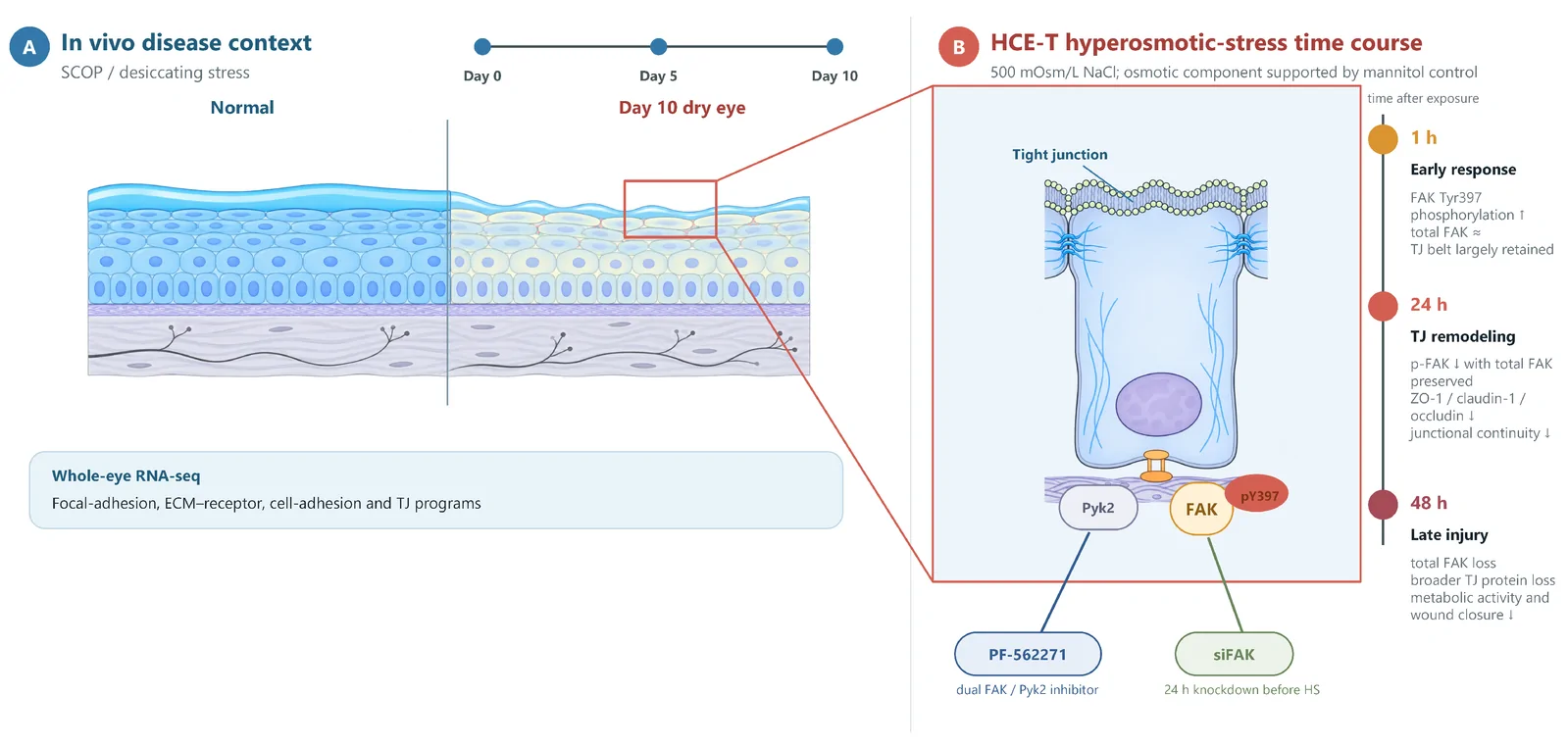
Evidence-based schematic of the time-dependent FAK-related response to hyperosmotic stress, with parallel in vivo disease context. (**A**) In vivo disease context: SCOP/desiccating-stress timeline from Day 0 to Days 5 and 10, contrasting the normal ocular surface with the Day-10 dry-eye phenotype; whole-eye RNA sequencing at Day 10 showed enrichment of focal-adhesion, extracellular-matrix–receptor, cell-adhesion, and tight-junction programs. (**B**) In vitro HCE-T time course: at 1 h, hyperosmotic stress increased FAK Tyr397 phosphorylation while total FAK remained comparatively stable; at 24 h, p-FAK decreased while total FAK remained comparatively preserved and ZO-1, claudin-1, and occludin abundance and junctional organization declined; at 48 h, loss of total FAK accompanied broader tight-junction-protein loss and cell injury. PF-562271 reduced FAK Tyr397 and Pyk2 Tyr402 phosphorylation and partially preserved tight-junction-protein abundance at 24 h, and pre-stress siRNA-mediated FAK knockdown partially preserved tight-junction-protein abundance and ZO-1 junctional continuity; the osmotic component is supported by the osmolality-matched mannitol control.

In this study, we asked how FAK-related signaling relates to the tight-junction protein alterations that accompany hyperosmotic stress at the ocular surface. In SCOP/desiccating-stress mice, whole-eye RNA sequencing showed coordinated remodeling of focal-adhesion, ECM–receptor, cell-adhesion, and tight-junction programs, pointing the subsequent experiments toward adhesion and junction biology. The HCE-T experiments then carried this theme to the protein level: FAK Tyr397 phosphorylation rose within 1 h of hyperosmotic exposure, fell by 24 h while total FAK was preserved, and gave way to loss of total FAK by 48 h as cells were injured. TJ protein abundance and junctional continuity declined along the same course, and both PF-562271 and FAK knockdown attenuated these changes (Figure 9). Together, these findings place FAK-related signaling within the hyperosmotic TJ protein response.

The time course itself is informative. A rapid Tyr397 phosphorylation response is characteristic of epithelial and fibroblast-like cells exposed to osmotic shrinkage [14,15]; its reversal by 24 h, with p-FAK falling while total FAK stays constant, is most consistent with dephosphorylation rather than protein loss. By 48 h, total FAK declines together with claudin-1, ZO-1, and occludin and with overt cell injury, so late p-FAK values partly reflect the state of the cells rather than signaling alone. Two technical observations fit this reading. The 24 h p-FAK/FAK ratio fell significantly in the inhibitor experiment (Figure 7B) and in the same direction, without reaching significance, in the independent dose–time-course experiment (Figure 6L); the difference in significance may reflect between-experiment variability rather than conflicting biology. Likewise, per-nucleus immunofluorescence detected the decline in total FAK earlier than immunoblotting did; differences in epitope exposure and in the separate staining batches used for each time point plausibly contribute, which is why IF comparisons were confined to within time points. At 1 h, immunoblotting detected lower total claudin-1 abundance at 500 mOsm/L, whereas the IF junctional-intersection metric did not show a corresponding significant reduction. These assays measure different endpoints—total cellular abundance versus the spatial continuity of junction-associated staining—and have different sensitivities; the early findings are therefore not directly interchangeable.

In the transcriptomics, the striking feature is coordination rather than magnitude. Pathway analysis retained all 3741 FDR-significant genes, of which only 823 also met the |log_2_FC| ≥ 1 cutoff, and the tight-junction program reached significance only in the larger set because most of its member genes changed by less than twofold. Whole-eye sampling explains the remaining terms: with retina, RPE, lens, conjunctiva, and cornea all contributing RNA, visual-perception, phototransduction, and lens-associated terms are tissue-composition signals; accordingly, they faded when a high-specificity retinal/RPE/lens marker set was excluded, while the focal-adhesion–ECM–cell-adhesion–TJ axis remained intact. We therefore use the transcriptomics as pathway context for the cell experiments, not as cornea-specific or transcript-to-protein evidence.

How FAK-family signaling couples to TJ protein loss remains open. Plausible intermediates—Src-family kinases recruited to phosphorylated Tyr397 [16], FAK–Src signaling through paxillin and MLCK during adhesion turnover [17], myosin-light-chain-phosphorylation-mediated tight-junction remodeling [18], and MMP-mediated proteolysis [6,7]—were not measured here and remain hypotheses. The direction of FAK influence is also tissue-dependent: FAK-associated complexes accompany junctional breakdown in cadmium-exposed testis [12], whereas in intestinal epithelium FAK supports tight-junction redistribution and barrier maintenance [13]. The corneal epithelial response described here—a brief phosphorylation burst followed by progressive TJ protein decline—adds a third case to this spectrum and cautions against assuming a single conserved role for FAK at junctions.

The inhibitor and knockdown experiments provide perturbational support for this association, each with defined caveats. PF-562271 lowered FAK Tyr397 phosphorylation and reduced Pyk2 Tyr402 phosphorylation, which hyperosmolarity alone did not significantly increase at 1 h; it preserved ZO-1, claudin-1, and occludin at 24 h, with stronger claudin-1 preservation at 250 nM–2.5 μM than at 10 nM, and the parallel effects of osmolality-matched mannitol indicate that osmolarity itself, rather than NaCl specifically, drives the response. PF-562271 inhibits Pyk2 as well as FAK, and additional off-target effects at 2.5 μM cannot be excluded. The CCK-8 signal also declined at higher concentrations, so the 2.5 μM findings must be read against a background of reduced metabolic activity. Specificity therefore rests chiefly on the siFAK experiments, in which knocking down FAK alone attenuated the loss of ZO-1, claudin-1, and occludin and partially preserved ZO-1 junctional continuity; an independent siRNA duplex and a rescue construct would complete the genetic evidence. The timing of the phosphorylation response suggests that temporal factors may be relevant to intervention design, but the continuous-exposure experiments do not establish a discrete early causal window. Time-of-addition or washout experiments would be required to resolve the temporal requirement for FAK signaling. Pathway specificity, barrier-function assays, in vivo efficacy, ocular formulation, and ocular-surface safety remain untested, and the present data do not support clinical use of PF-562271.

The synchronized time course of FAK Tyr397 phosphorylation and progressive TJ protein loss under hyperosmotic stress described here is consistent with a contribution of FAK-related signaling to progressive cell–cell junction-protein alterations and reduced repair capacity in the corneal epithelium. Attenuation of TJ protein loss by pharmacological FAK/Pyk2 inhibition and genetic FAK knockdown supports further investigation of FAK-related signaling as a candidate approach to preserving corneal epithelial junctional protein abundance. The timing of the phosphorylation response suggests that treatment timing may be relevant, but the present experiments do not define an early causal window or an effective therapeutic schedule. These translational implications await validation in future in vivo functional studies and clinical research.

Several limitations remain. First, no transepithelial electrical resistance or paracellular-flux assay was performed, so functional barrier impairment or recovery was not demonstrated. Second, corneal FAK, p-FAK, and TJ proteins could not be examined in vivo because no tissue remained after the planned analyses; the animal data are limited to phenotype, histology, immunohistochemistry, and whole-eye transcriptomics. Third, no time-of-addition or washout experiment was performed, so the continuous inhibitor and siRNA designs do not define a discrete causal time window for FAK signaling. Fourth, PF-562271 inhibits both FAK and Pyk2; additional off-target effects at 2.5 μM cannot be excluded, and higher concentrations reduced the CCK-8 signal. A matched four-group factorial CCK-8 assessment under isotonic and hyperosmotic conditions was not performed, and ZO-1/claudin-1 immunofluorescence was not repeated specifically in the inhibitor groups. Fifth, wide-field single-marker imaging does not demonstrate p-FAK/ZO-1 colocalization; future confocal dual-label imaging is required. Although FAK/p-FAK IOD was normalized per DAPI-positive nucleus, separate field-level cell-density data were not displayed. Sixth, proliferation was not blocked in the scratch assay, and immunoblot normalization relied on β-actin rather than total-protein staining; the stability of β-actin across experimental conditions was not independently verified. Finally, the animal and cell experiments were exploratory and modestly sized, and no formal a priori power calculation was performed; only female mice and an immortalized HCE-T line were used, the whole-eye RNA-seq data are not cornea-specific, 450–500 mOsm/L represents a high experimental stress relative to typical tear values, and tear osmolality was estimated by controlled dilution with solute-conservation back-calculation, which may amplify sampling-volume and instrument-reading errors.

## 4. Materials and Methods

### 4.1. Dry Eye Model Establishment

Female SPF-grade C57BL/6J mice (6–8 weeks old, 16–18 g) were purchased from Suzhou SBF Biotechnology Co., Ltd. (Suzhou, China; Animal License No. SCXK (Zhe) 2024-0004) and housed at the China Pharmaceutical University animal facility under SPF conditions (20–24 °C; 12 h light/12 h dark cycle; no more than four mice/cage). Only female mice were used to maintain a uniform exploratory cohort and because DED is more prevalent in women [1]; sex was not evaluated as a biological variable. All procedures were approved by the Experimental Animal Ethics Committee of China Pharmaceutical University (YSL-202506086; 19 June 2025). After 7 days of acclimatization, animals were assigned by random-number table to PBS vehicle-control and SCOP/desiccating-stress conditions. The main phenotyping/histology cohort comprised nine mice per condition. Six of the nine SCOP mice contributed longitudinal measurements from both eyes on Days 0, 5, and 10 (*n* = 6 mice at each time point); within-mouse handling of the bilateral readings is specified in Section 4.3, Section 4.4, Section 4.5 and Section 4.6. Terminal hematoxylin–eosin analysis used one right-eye cornea from each of all nine mice per condition (*n* = 9 corneas/group). Immunohistochemistry used one right-eye cornea from a three-mouse subset of these same nine mice per condition (*n* = 3 corneas/group). Repeated sections or fields within a cornea were treated as subsamples rather than additional biological replicates. Each terminal cornea-level value therefore represented one mouse. The independent RNA-seq cohort is described in Section 4.2. The sample size was based on preliminary effects; no formal a priori power calculation was performed. The model cohort received subcutaneous injections of (−)-scopolamine hydrobromide trihydrate (98%; Beyotime Biotechnology, Shanghai, China; catalog no. Y207606) at 2.5 mg/mL in PBS (0.1 mL per injection) at 08:00, 12:00, 16:00, and 20:00 daily and was housed at 30–40% relative humidity; the vehicle-control cohort received matched subcutaneous PBS injections (0.1 mL/injection) at the same four daily time points and was housed at 50–60% relative humidity, with other conditions matched. Animals were monitored daily, and no unexpected deaths or exclusions occurred. All terminal analyses were performed on Day 10.

### 4.2. Transcriptome Sequencing and Analysis

RNA sequencing used an independent cohort of three additional mice per condition that did not overlap with the nine-mouse phenotyping/histology cohort described in Section 4.1. Thus, 12 mice were used per condition across the animal experiments (24 mice in total). Both whole eyeballs from each euthanized SCOP/model (M) or PBS vehicle-control (NC) mouse were collected on Day 10 and pooled within each mouse to generate one biological replicate (*n* = 3 mice/group). Samples were snap-frozen in liquid nitrogen and stored at −80 °C. Total RNA was extracted with MagZol reagent (Magen Biotechnology Co., Ltd., Guangzhou, China); RNA concentration and purity were assessed using a NanoDrop 2000 spectrophotometer (Thermo Fisher Scientific, Waltham, MA, USA), and RNA integrity was verified by agarose gel electrophoresis and analysis with an Agilent 5300 Fragment Analyzer (Agilent Technologies, Santa Clara, CA, USA). Library preparation and sequencing were performed by Shanghai Majorbio Bio-pharm Biotechnology Co., Ltd. on the DNBSEQ-T7 platform (MGI Tech Co., Ltd., Shenzhen, China). For the six M and NC libraries analyzed in this study, quality filtering retained 36.62–45.86 million clean reads per library; Q30 values were 93.99–94.86%, GC contents were 49.54–50.41%, total mapping rates were 96.32–96.83%, and unique mapping rates were 91.60–93.02%. Raw reads were filtered with fastp, aligned to the mouse GRCm39 genome with HISAT2, and quantified with RSEM. Principal-component analysis was performed on transformed expression values to examine sample-level separation.

DESeq2 was used for differential-expression analysis. Strict large-effect DEGs were defined as FDR < 0.05 and |log_2_FC| ≥ 1 for gene-level reporting. For pathway enrichment, all genes meeting FDR < 0.05 were analyzed irrespective of fold-change magnitude to retain coordinated moderate-effect signals. GO and KEGG over-representation analyses were performed with clusterProfiler using ontology/database-specific annotated gene universes and Benjamini–Hochberg correction. All genes with nominal *p* < 0.05 (*n* = 4979), without application of an FDR threshold, were used only as a threshold-sensitivity reference. In the mutually exclusive volcano-plot categories, “nominal-only” denotes genes with nominal *p* < 0.05 but FDR ≥ 0.05 (*n* = 1238).

Hypothesis-driven TJ analyses used database-defined TJ gene sets and an independently frozen epithelial TJ core; mROAST provided mixed and directional self-contained tests, and CAMERA provided a complementary competitive test. To assess the effect of whole-eye tissue composition, enrichment was repeated after excluding a prespecified high-specificity retinal/RPE/lens marker set from the FDR-significant pathway foreground. This sensitivity analysis did not redefine the primary gene-level results.

### 4.3. Tear Production Tests

Phenol-red threads were placed sequentially in the lower conjunctival sac of both eyes for 15 s each. The wetted length was measured in triplicate in each eye; the two within-eye means were averaged to yield one value per mouse. The mouse, not the repeated reading, was the statistical unit; assessors were blinded to group allocation.

### 4.4. Tear-Film Break-Up Time

Fresh 0.5% (*w*/*v*) fluorescein sodium in PBS was instilled into the lower conjunctival sac of both eyes without anesthesia. After three blinks, tear-film break-up time (TBUT) was recorded under a slit-lamp microscope (SL-2G, Topcon Corporation, Tokyo, Japan) as the interval to the first dark spot. Three readings were averaged within each eye, and the two eyes were averaged within each mouse; assessors were blinded to group allocation.

### 4.5. Tear Osmolality Measurement

Tear osmolality was estimated without anesthesia by a controlled-dilution, freezing-point method. Tears were collected from the inferior tear meniscus of both eyes with a calibrated glass capillary, avoiding contact with the corneal and conjunctival surfaces and without globe compression to minimize reflex tearing; tears from both eyes were pooled as one biological sample per mouse. The recovered tear volume (V_t_; approximately 1–2 μL) was recorded. Sterile saline with independently measured osmolality O_s_ was added to a final volume of 20 μL, and the diluted sample osmolality O_m_ was measured by freezing-point depression (OM819M osmometer, Vogel MedTec GmbH, Fernwald, Germany), calibrated before each session. Original tear osmolality was estimated by solute conservation as O_t_ = [O_m_(20 μL) − O_s_(20 μL − V_t_)]/V_t_ and is expressed in mOsm/kg H_2_O. These values represent estimates obtained after controlled dilution and back-calculation rather than direct undiluted measurements; this limitation is acknowledged in the Discussion. The same mice were measured on Days 0, 5, and 10, and each mouse was an independent biological replicate analyzed with repeated-measures models. The endpoint was analyzed as a continuous measure; no human diagnostic cutoff was used to define model success, and the investigator was blinded to group allocation.

### 4.6. Corneal Fluorescein Staining Score

Both eyes were assessed after instillation of 0.5% fluorescein sodium. After three blinks and a 5 min interval, each cornea was examined under cobalt-blue illumination and divided into five regions. Each region was scored from 0 to 3 using the modified NEI approach [19], yielding a total CFS score of 0–15 per eye; the two eyes were averaged within each mouse.

### 4.7. Hematoxylin–Eosin Staining

Corneas were fixed, paraffin-embedded, and sectioned at 4 μm. Sections were deparaffinized, rehydrated, stained with hematoxylin for 5 min and eosin for 1 min, dehydrated, cleared, and mounted. Central-corneal epithelial thickness and epithelial-cell number per 100 μm were measured in ImageJ 1.54p (Fiji distribution; National Institutes of Health, Bethesda, MD, USA) by an observer blinded to group. Measurements from sections and fields were averaged within each right-eye cornea; nine corneas from nine mice were analyzed per condition (*n* = 9 corneas/group), and the cornea-level value was the statistical unit.

### 4.8. Immunohistochemistry

Sections were incubated with 3% H_2_O_2_, blocked with normal goat serum, and incubated overnight at 4 °C with anti-MMP9 (1:200; AF5228, Affinity Biosciences, Changzhou, China) or anti-MMP3 (1:100; 380816, ZenBio, Chengdu, China). Signals were developed with DAB and counterstained with hematoxylin. MMP3 and MMP9 staining were quantified in ImageJ as average optical density (AOD) using identical color-deconvolution and threshold settings within each marker. Field-level values were averaged within each right-eye cornea; three corneas from three mice were analyzed per condition (*n* = 3 corneas/group) by a blinded observer, and the cornea-level value was the statistical unit.

### 4.9. Hyperosmotic Stress Treatment

The SV40-immortalized human corneal epithelial cell line HCE-T [20] was obtained from the Cell Bank, Type Culture Collection, Chinese Academy of Sciences (Shanghai, China; catalog no. GNHu67). Cell identity was authenticated by short tandem repeat (STR) profiling; the resulting profile was consistent with the DNA profiles reported by ExPASy and showed no evidence of contamination by other human cell lines. Mycoplasma testing by PCR/qPCR was negative. Cells were maintained in DMEM/F12 (Gibco, Thermo Fisher Scientific, Waltham, MA, USA) supplemented with 6% fetal bovine serum, 1% penicillin–streptomycin, 5 μg/mL insulin, and 10 ng/mL human epidermal growth factor. Experiments used logarithmically growing cells three to five passages after thawing. Hyperosmotic treatment was initiated when cultures were approximately 80% confluent, except for scratch assays, which used confluent monolayers as described in Section 4.12. Hyperosmotic media were prepared by adding sterile NaCl to 312 mOsm/L control medium and were verified with a freezing-point osmometer. Morphology/detachment assays used 312, 400, 450, 500, 550, and 600 mOsm/L; CCK-8 assays used 312, 350, 400, 450, 500, and 550 mOsm/L; scratch assays, immunofluorescence, and immunoblotting used 312, 350, 400, 450, and 500 mOsm/L. Cells were analyzed after 1, 24, or 48 h as specified. A 500 mOsm/L mannitol condition was included in the PF-562271 concentration-response experiment as an osmolality-matched non-ionic control.

### 4.10. Cell Morphology Observation and Cell-Detachment Assay

HCE-T cells were seeded in six-well plates (5 × 10^4^ cells/well) and exposed to the indicated media after attachment. Bright-field morphology was imaged with a EVOS FLoid system (Thermo Fisher Scientific, Waltham, MA, USA) using a 20× objective under fixed settings (scale bar, 125 μm). Three non-overlapping fields per condition were analyzed in ImageJ by a blinded investigator. Detachment rate was the threshold-defined detached area divided by total field area; fields were averaged to one value per independent experiment.

### 4.11. Cell-Viability Assay

For hyperosmotic-stress experiments, HCE-T cells were exposed to 312–550 mOsm/L medium for 1, 24, or 48 h. CCK-8 reagent (Beyotime Biotechnology, Shanghai, China) was added according to the manufacturer’s instructions, and absorbance at 450 nm was measured with a Multiskan FC microplate photometer (Thermo Fisher Scientific, Waltham, MA, USA). Technical wells were averaged, and values were normalized to the 312 mOsm/L condition at the corresponding time point. PF-562271 dose-related metabolic viability was assessed after 24 h at 0.01, 0.25, 1, 2.5, 5, 10, or 15 μM.

### 4.12. Wound-Healing Assay

Confluent HCE-T monolayers were scratched with a sterile 10 μL pipette tip, washed three times with PBS, and incubated in serum-free medium at the indicated osmolarities for the remainder of the assay. Images were acquired at 0, 24, and 48 h using a Leica DMI3000B microscope (Leica Microsystems CMS GmbH, Wetzlar, Germany) (5× objective; 50× total magnification; scale bar, 500 μm). Wound closure was calculated in ImageJ as [(initial width − width at the indicated time)/initial width] × 100%. Mitomycin C was not used, and proliferation was not quantified; therefore, the assay was interpreted as wound closure rather than migration alone. Three independent experiments were performed, with technical fields averaged within each experiment.

### 4.13. Immunofluorescence Staining

HCE-T cells (5 × 10^4^ cells/well) were seeded on sterile coverslips, allowed to attach for 24 h, and exposed to hyperosmotic medium for 1, 24, or 48 h. Cells were washed, fixed in 4% paraformaldehyde, permeabilized with 0.1% Triton X-100, and blocked with 5% BSA.

Cells were incubated overnight at 4 °C with antibodies against ZO-1 (1:200; 21773-1-AP, Proteintech Group, Inc., Rosemont, IL, USA), claudin-1 (1:200; 13050-1-AP, Proteintech), FAK (1:200; 12636-1-AP, Proteintech), or p-FAK Tyr397 (1:200; 83933-1-RR, Proteintech), followed by Alexa Fluor 488-conjugated goat anti-rabbit IgG (1:200; A0423, Beyotime) and DAPI-Fluoroshield (P0131-5 mL, Beyotime). Images were acquired on an Olympus BX43 wide-field fluorescence microscope (Olympus Corporation, Tokyo, Japan). Within each marker and time-point batch, exposure, gain, magnification, and display processing were held constant; different time points were processed in separate sessions and were not compared by absolute intensity. Three non-overlapping fields were sampled per condition in each independent experiment.

For FAK and p-FAK, background-corrected IOD was calculated for each field and divided by the number of DAPI-positive nuclei in that field. The three fields were averaged to one value per independent experiment (*n* = 3). No IF p-FAK/FAK ratio was calculated because the two targets were measured on different coverslips. For ZO-1 and claudin-1 organization, a fixed Python pipeline (Python 3.8.2; OpenCV 4.11.0; NumPy 1.23.5; SciPy 1.10.1) applied background correction, percentile-based normalization, and 80 nested rectangular contours. Fluorescence peaks at contour–junction intersections were detected with marker-specific parameters held constant across groups and normalized to total contour length as intersections per 1000 contour pixels. Fields were averaged to one experiment-level value (*n* = 3). Analyses were performed blinded to group labels.

### 4.14. Immunoblot Analysis

HCE-T lysates were prepared in RIPA buffer containing protease and phosphatase inhibitors and quantified by BCA assay (P0010, Beyotime). Equal protein amounts were separated by SDS-PAGE, transferred to PVDF membranes, blocked with 5% BSA, and incubated overnight at 4 °C with primary antibodies against ZO-1 (1:2000; 21773-1-AP, Proteintech), occludin (1:2000; 13409-1-AP, Proteintech), claudin-1 (1:2000; 13050-1-AP, Proteintech), FAK (1:2000; 12636-1-AP, Proteintech), p-FAK Tyr397 (1:2000; 83933-1-RR, Proteintech), Pyk2 (1:2000; 17592-1-AP, Proteintech), or p-Pyk2 Tyr402 (1:2000; 44-618G, Invitrogen, Thermo Fisher Scientific, Waltham, MA, USA), together with β-actin (1:6000; 20536-1-AP, Proteintech) as loading control. The FAK antibody 12636-1-AP produced an observed band at approximately 110 kDa (manufacturer-calculated molecular weight, 119 kDa). After HRP-conjugated secondary antibody incubation (1:5000; RGAR001, Proteintech), signals were detected using ECL (89901, Thermo Fisher) on a ChemiDoc XRS+ system (Bio-Rad Laboratories, Hercules, CA, USA) and quantified in ImageJ. Total FAK and p-FAK, and total Pyk2 and p-Pyk2, were detected on parallel gels loaded with the same BCA-quantified lysates; phosphorylated-to-total ratios were normalized within the corresponding experiment. Figure 6 comprised three independent biological experiments, whereas Figure 7 and Figure 8 comprised three.

### 4.15. Pharmacological Inhibition of FAK/Pyk2 Signaling

PF-562271 was treated as a dual FAK/Pyk2 inhibitor [21]. For four-group experiments, cells were pretreated with 2.5 μM PF-562271 or vehicle for 1 h and then transferred to 312 or 500 mOsm/L medium; inhibitor or vehicle was maintained throughout the subsequent 1 or 24 h exposure. Immunoblotting assessed total FAK, p-FAK Tyr397, total Pyk2, p-Pyk2 Tyr402, ZO-1, claudin-1, and occludin as specified in Figure 7.

For concentration-response studies, cells were assigned to 312 mOsm/L medium, 500 mOsm/L NaCl medium, osmolality-matched 500 mOsm/L mannitol medium, or 500 mOsm/L NaCl medium containing 10 nM, 250 nM, or 2.5 μM PF-562271. The p-FAK/FAK ratio was measured at 1 h and claudin-1 at 24 h. Separately, CCK-8 metabolic viability was assessed after 24 h exposure to 0.01–15 μM PF-562271. Each experiment comprised three independent biological replicates.

### 4.16. siRNA-Mediated FAK Knockdown

HCE-T cells were transfected with a vendor-supplied non-targeting control siRNA (siCtrl; d-NC) or the FAK-targeting duplex hFAK si-1 (siFAK1; shown as siFAK in Figure 8; KeyGEN BioTECH, Nanjing, China) at a final concentration of 50 nM using Lipofectamine 2000 (Invitrogen, Thermo Fisher Scientific, Waltham, MA, USA) according to the supplier’s protocol. For each well of a six-well plate, 5 μL of 20 μM siRNA stock and 6 μL of Lipofectamine 2000 were separately diluted in Opti-MEM, combined for 20 min at room temperature, and added to antibiotic-free medium to a final volume of 2 mL. The siCtrl sequences were 5′-UUCUCCGAACGUGUCACGUdTdT-3′ (sense) and 5′-ACGUGACACGUUCGGAGAAdTdT-3′ (antisense). The siFAK1 sequences were 5′-GCCCAGGUUUACUGAACUUAATT-3′ (sense) and 5′-UUAAGUUCAGUAAACCUGGGCTT-3′ (antisense). After 4–6 h, the transfection medium was replaced with complete culture medium. At 24 h after transfection, cells were exposed to 312 or 500 mOsm/L medium for an additional 24 h. Knockdown efficiency and ZO-1, claudin-1, and occludin abundance were assessed using immunoblotting, and ZO-1 organization was evaluated by immunofluorescence and the junctional-intersection pipeline described above. Three independent experiments were performed.

### 4.17. Statistical Analysis

Statistical analyses were performed in GraphPad Prism 10.0 (GraphPad Software, Boston, MA, USA), and data are presented as mean ± SEM with individual biological replicates shown. For Figure 1C–F, the same mice were measured longitudinally and analyzed using repeated-measures one-way ANOVA followed by Tukey’s multiple-comparisons test (complete data at all time points; Greenhouse–Geisser correction left all conclusions unchanged). Cornea-level two-group comparisons used two-sided unpaired *t* tests, with *n* = 9 right-eye corneas from nine mice per group for Figure 1J,K and *n* = 3 right-eye corneas from three mice per group for Figure 1L,M. For cell experiments, technical wells or fields were averaged within each independent experiment; osmolarity groups were then compared within each time point by one-way ANOVA followed by Tukey’s multiple-comparisons test. Absolute IF intensities were not compared across independently stained time-point batches. With *n* = 3, formal normality tests were not used to determine test choice because they have limited information; parametric analyses were prespecified for continuous normalized outcomes in balanced designs, and results are considered exploratory. RNA-seq differential expression used DESeq2 with Benjamini–Hochberg FDR control. A two-sided *p* < 0.05 was considered statistically significant.

## 5. Conclusions

Hyperosmotic stress elicited a time-dependent FAK response in HCE-T cells, with early Tyr397 phosphorylation, reduced phosphorylation at preserved total FAK by 24 h, and later loss of total FAK accompanying cell injury. These changes coincided with reduced abundance and continuity of TJ proteins. Whole-eye transcriptomics provided an FDR-controlled adhesion/TJ pathway context. PF-562271 and siRNA-mediated FAK knockdown each partially attenuated TJ protein changes, supporting involvement of FAK-related signaling while leaving FAK-versus-Pyk2 specificity unresolved for the pharmacological data. The findings identify a candidate mechanism for functional and in vivo validation.

## Data Availability

The RNA-sequencing data have been deposited in the NCBI Gene Expression Omnibus under accession GSE351029. Other raw data supporting this study are available from the corresponding author upon reasonable request.

## Supplementary Materials

The following supporting information can be downloaded at: https://www.mdpi.com/article/10.3390/ijms27219716/s1.

## Abbreviations

The following abbreviations are used in this manuscript:

DED	Dry Eye Disease
FAK	Focal Adhesion Kinase
p-FAK	Phosphorylated Focal Adhesion Kinase
TJ	Tight Junction
HCE-T	Human Corneal Epithelial Cell Line
SCOP	Scopolamine Hydrobromide
CFS	Corneal Fluorescein Staining
TBUT	Tear Break-Up Time
HE	Hematoxylin–Eosin
IHC	Immunohistochemistry
IOD	Integrated Optical Density
SEM	Standard Error of the Mean
DEGs	Differentially Expressed Genes
MMP	Matrix Metalloproteinase
Pyk2	Proline-Rich Tyrosine Kinase 2
PF-562271	Dual FAK/Pyk2 Inhibitor
AOD	Average Optical Density
FDR	False Discovery Rate
PCA	Principal-Component Analysis
