# Supplementary material for: Rapid FAK Phosphorylation and Progressive Tight-Junction Protein Alterations Under Hyperosmotic Stress in Corneal Epithelial Cells
Source: Int J Mol Sci. 2026 Oct 29;27(21):9716. doi: 10.3390/ijms27219716 (PMC13763085; doi:10.3390/ijms27219716)
Supplement: Supplementary file 1 [file ijms-27-09716-s001.zip › Figure_S1_Legend.pdf]

# Supplementary Figure Legend

**Figure S1.** Sensitivity and targeted analyses of the whole-eye transcriptomic data. **(A)** Threshold sensitivity of tight-junction pathway enrichment across strict large-effect differentially expressed genes (DEGs; $n$ = 823), all false-discovery-rate (FDR)-significant genes ($n$ = 3741), and all nominally significant genes (nominal $p < 0.05$; $n$ = 4979). Bubble color and size denote $-\log_{10}$(conservative FDR) and the number of pathway-hit genes, respectively. **(B)** Multiple database-defined tight-junction gene sets were evaluated using self-contained mROAST and complementary competitive CAMERA tests. Significant mixed-direction remodeling was detected across several sets, whereas consistent directional suppression was not observed across all sets. Filled red and open circles denote FDR < 0.05 and FDR ≥ 0.05, respectively; circle size denotes $-\log_{10}$(FDR), and numeric labels give FDR values. **(C)** $\text{Log}_2$ fold-change ($\log_2$FC) and FDR values for prespecified focal-adhesion and tight-junction genes, including *Tjp1*, *Ocln*, *Src*, and *Cldn1*. Filled and open symbols denote DESeq2 and transcripts-per-million (TPM)-derived estimates, respectively; colors indicate functional modules. **(D)** Pathway enrichment before and after exclusion of a frozen high-specificity retinal/retinal pigment epithelium (RPE)/lens marker set. Red points indicate the complete FDR-significant foreground ($n$ = 3741), and blue points indicate the marker-excluded foreground ($n$ = 3709). M, SCOP/desiccating-stress model group; NC, phosphate-buffered-saline vehicle-control group; SCOP, scopolamine hydrobromide; FDR, false discovery rate; KEGG, Kyoto Encyclopedia of Genes and Genomes; GO, Gene Ontology; TJ, tight junction; ECM, extracellular matrix; RPE, retinal pigment epithelium; TPM, transcripts per million; NS, not significant. Here, $n$ denotes the number of genes in the indicated foreground. mROAST is a self-contained rotation gene-set test, whereas CAMERA is a competitive gene-set test that accounts for inter-gene correlation.
